# Antithrombotic Treatment After Left Atrial Appendage Occlusion in Older Atrial Fibrillation Patients

**DOI:** 10.1093/geroni/igaf122.3384

**Published:** 2025-12-31

**Authors:** Xiaojuan Liu, Sebastian Schneeweiss, Daniel Singer, Jerry Avorn, E Kevin Heist, Joshua Lin

**Affiliations:** Brigham and Women’s Hospital, Boston, Massachusetts, United States; Brigham and Women’s Hospital, Boston, Massachusetts, United States; Massachusetts General Hospital, Boston, Massachusetts, United States; Brigham and Women’s Hospital, Boston, Massachusetts, United States; Massachusetts General Hospital, Boston, Massachusetts, United States; Brigham and Women’s Hospital, Boston, Massachusetts, United States

## Abstract

Left atrial appendage occlusion (LAAO) is an alternative to oral anticoagulants (OAC) for stroke prevention in nonvalvular atrial fibrillation (NVAF); however, real-world data on adherence to its postprocedural treatment protocol remain limited. We evaluated post-LAAO antithrombotic treatment patterns in a cohort of 16304 NVAF patients (mean [SD] age = 78 [6]; 47% female; CHA2DS2-VASc score = 5.0 [1.5]) undergoing first-time LAAO using Optum claims data 2015-2024. OAC discontinuation was defined as a ≥ 60-day gap in supply, and prolonged OAC use was operationally defined as either (1) an OAC refill following a TEE performed 30–90 days post-implantation or (2) OAC refill beyond 90 days post-implantation, regardless of TEE completion. Among LAAO recipients, 9844 (60%) received concomitant OAC and 3499 (10%) received P2Y12 inhibitor at implantation. Transesophageal echocardiography (TEE) was performed in 11237 (69%) patients at 45 (±15) days. Among OAC users (n = 9844), 30% and 85% discontinued OAC by 45 days and 6 months post-implantation, respectively; only 23% patients followed the standard postprocedural protocol. Common deviation from the protocol included no TEE at 45 (±15) days (30%), early OAC discontinued (22%), continued OAC use post-TEE (10%), and P2Y12 inhibitor initiated early or not initiated (10%). Prolonged OAC use was observed in 1970 patients. Factors associated with a higher likelihood of prolonged OAC use included older age, Black race, cardio ablation, and a longer duration of prior OAC use. These findings suggest moderate to low adherence to the LAAO postprocedural protocol, warranting further evaluation of the clinical impact of these adherence patterns.

